# Treatment of a High-risk Thoracolumbar Compression Fracture Using Bilateral Expandable Titanium SpineJack Implants

**DOI:** 10.7759/cureus.4701

**Published:** 2019-05-21

**Authors:** Jason Hartman, Michelle Granville, Robert E Jacobson

**Affiliations:** 1 Pain Medicine, Larkin Community Hospital, Miami, USA; 2 Neurological Surgery, University of Miami Hospital, Miami, USA

**Keywords:** balloon kyphoplasty, vertebral augmentation, thoraco-lumbar fracture, spinejack, vertebral height restoration

## Abstract

In this case, an 80-year-old active patient developed an acute osteoporotic fracture after a fall at L1 above a previous interlaminar implant at L4-5 for stenosis with neurogenic claudication. Radiologic studies found both intra-discal and intra-vertebral vacuum clefts that are highly correlated with instability and progressive kyphosis. Long-term experience with kyphoplasty has shown that acute and subacute fractures can often be re-expanded; however, over three months to one year, the correction is frequently lost and the vertebral height continues to decrease leading to increased risk of both continued deformity and especially adjacent level fractures. The use of newly available titanium intra-vertebral implants combined with bone cement restores and maintains vertebral height and correction of deformities. Long-term studies also demonstrate a reduced risk of adjacent level fractures compared to balloon kyphoplasty. Using vertebral body implants that remain in place within the fractured vertebral body the initial height correction can be better maintained leading to less adjacent level fractures.

## Introduction

Thoraco-lumbar osteoporotic compression fractures have the highest risk of continued collapse because of their location at the mobile junction between the thoracic and lumbar spine which has the largest degree of motion of the spine [1]. Fractures in this area are also seen frequently after previous lumbar fusion or stabilization, as in this patient, with a previous L4-5 stabilization device [2]. A number of studies have found that patients with intradiscal and intra-vertebral vacuum clefts on computed tomography (CT) or magnetic resonance imaging (MRI), as in this patient, are also at a significantly higher risk of continued collapse even with kyphoplasty (KP) and especially if the cleft is not filled with cement at the time of the procedure [3-4]. If the patient fails conservative treatment including rigid bracing, indicated by persistent pain or vertebral collapse and progressive spinal deformity, options include vertebroplasty (VP), balloon kyphoplasty (BKP) or multi-segmental screw fixation, although the later can be problematic in elderly patients with co-morbidities and associated vertebral osteoporosis, which makes screw fixation difficult to maintain [5-6]. Subsequent kyphotic deformity, in turn, is thought to lead to shifting of the center of gravity more anteriorly, leading to an increased incidence of adjacent level fractures [7-10]. Paradoxically, it is been noted in some studies that there is a higher incidence of adjacent level fractures with BKP compared to VP, which is thought to be related to the increased density and biomechanical stresses of large amounts of bone cement, polymethylmethacrylate (PMMA), on the adjacent osteoporotic vertebra [10-11]. The thoracolumbar junction is also found to be more vulnerable to gradual height decrease and progressive deformity despite either VA or BKP [12]. Recent experience with using bilateral titanium implants, the SpineJack^R^ system (Stryker, Kalamazoo, Michigan, USA), that maintain the correction supplemented by use of lesser amounts of bone cement is a new option in these cases since the SpineJack (SJ) has been shown to both maintain the correction over one year studies and more importantly, has significant less incidence of adjacent level fractures [13]. This case highlights the use of the SJ implants for an L1 fracture because of all the above factors. 

## Case presentation

**Figure 1:**
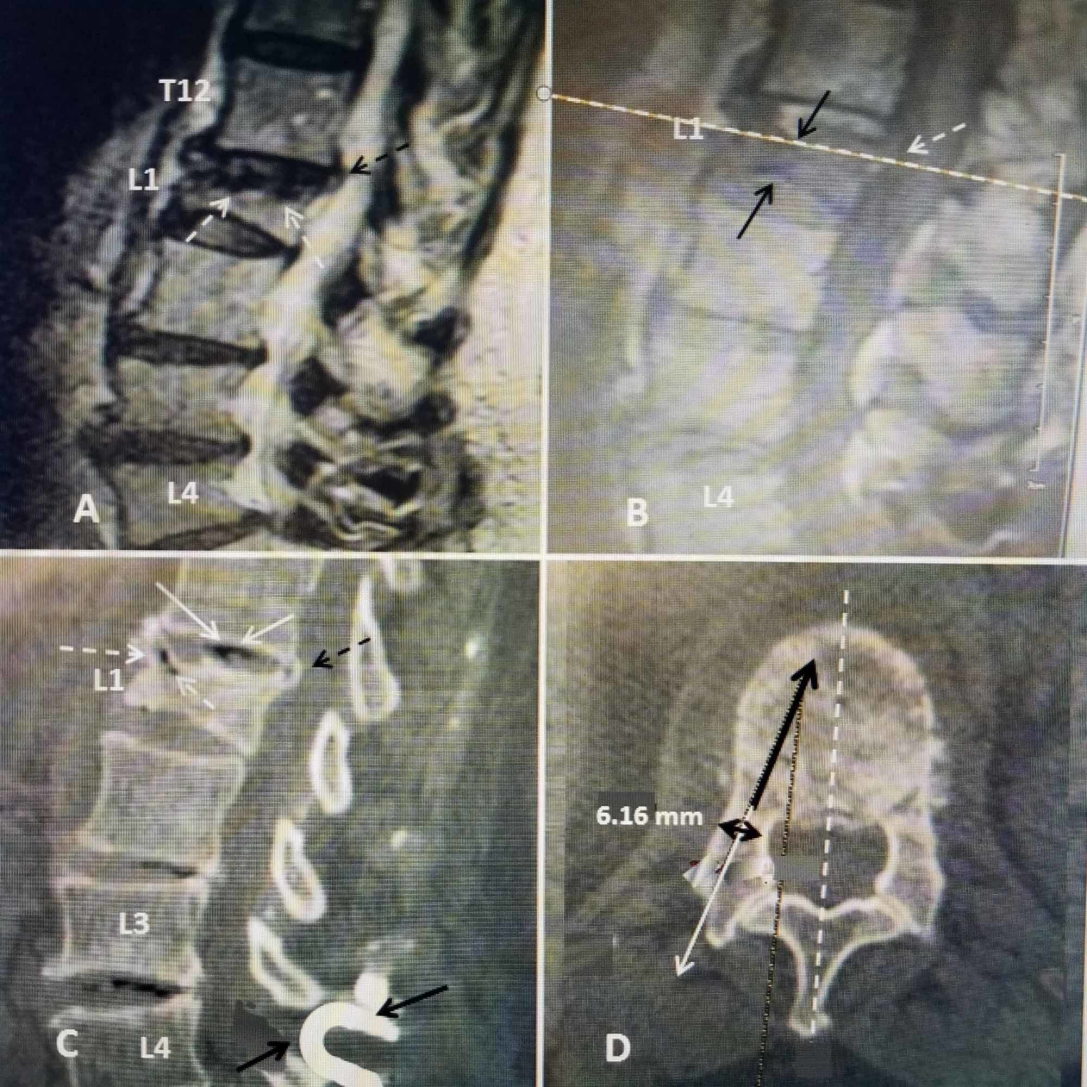
Pre-operative films of acute osteoporotic compression fracture at L1 A: Initial sagittal T2 MRI showing over 50% collapse of the L1 middle and anterior vertebral body (dashed white arrows) with minimal 2-mm displacement of the posterior part of the superior endplate (dashed black arrow) into the spinal canal. B: Sagittal T1 image showing there is also indentation of the inferior endplate of L1 causing biconcave compression (solid black arrows) and edema in the vertebral marrow. The cut used to measure for the SpineJack^R^ implant is indicated (dashed white line across scan). The 2-mm posterior superior endplate displacement is seen (dashed white arrows). C: Sagittal CT vacuum change within the T12-L1 disc space (solid white arrows) and also within the anterior superior part of the vertebral body under the superior endplate (dashed white arrow). Minimal 2-mm posterior displacement of the superior endplate is seen into the spinal canal (dashed black arrow). The previously inserted Coflex interlaminar device inserted at L4-5 is seen below the fracture (solid black arrows). D: Axial CT view showing the measurement of angle and depth for the implant (solid black arrow). By visualizing the line posteriorly (solid white line with arrow) the trajectory for insertion of the SpineJack^R^ is determined. The internal width (bicortical dimension) of the pedicle is indicated as 6.16 mm (double-headed black arrow) to select the best-closed implant size. The implant is designed to be positioned in front of the pedicle, posteriorly and just off-center of the midline anteriorly bilaterally (dashed white arrow).

The patient is a very active 80-year-old female who had a Coflex^R ^(Paradigm Spine, New York, NY) interlaminar implant for neurogenic claudication one year previously. She had returned to full activities including driving and exercise classes. Three weeks prior to the evaluation, the patient tripped while walking outside and fell on her back and had acute upper lumbar pain. After two weeks of persistent pain and taking anti-inflammatory and pain medication, she went to her primary physician who ordered X-rays and MRI. She was diagnosed with an acute L1 compression fracture with approximately 60% collapse anteriorly and early kyphotic deformity. She had percussion tenderness in the midline upper lumbar spine, paraspinal bilateral muscle spasms without lower extremity radiation. CT scans were also made for further study of the fracture and posterior wall, and to make measurements for use of the SJ implant. Measurements were made of the transverse width of the pedicle and the angled depth of the vertebra anterior to the pedicle on both sides to determine the size for selecting the correct implant that could be placed through the pedicle, similar to the placement of pedicle screws (Figure 1).

**Figure 2:**
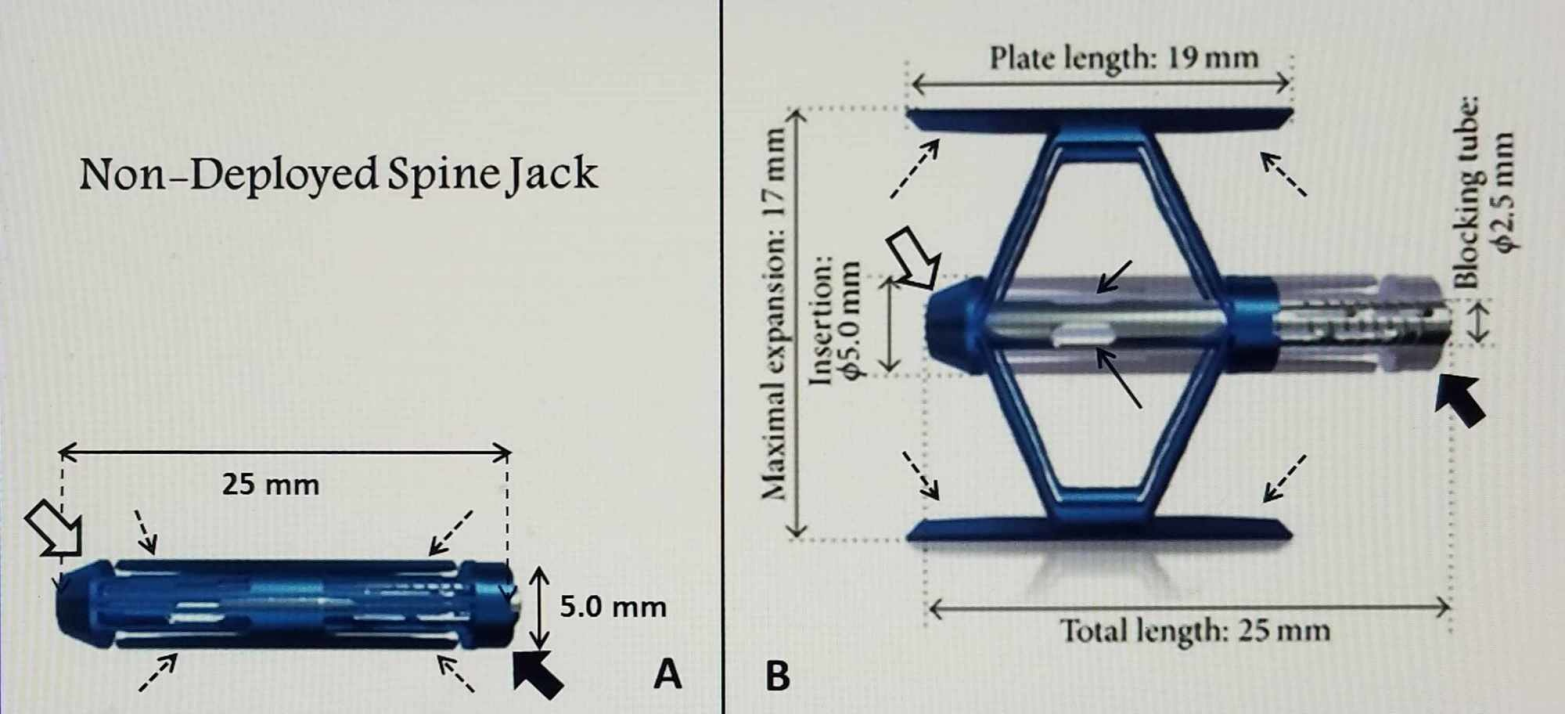
Example of a closed compared to a fully deployed SpineJack implant A: A closed 5.0-mm SpineJack with the undeployed 'struts' (fine black dashed arrows) that are recessed between the insertion tip (open black arrow) and the blocking tube (solid large black arrow). The delivery instrument attaches to the blocking tube. B: Opened and deployed 5.00 mm SpineJack showing length and height dimensions when fully opened. The total length of the opened implant, including insertion connection, is slightly longer than the deployed struts (dashed black arrows). The implants are sized using reconstructed CT and/or MRI scans for each patient. The insertion tip (open large white arrow) and blocking connection where the insertion instrument is attached (solid large black arrow) can be identified. The side ports where cement exits after being injected through the implant is seen (solid black arrows). For maximum endplate support and re-expansion, it is critical to center the struts under the depressed endplate. The total length of the implant, including the insertion port and blocking tube, is positioned within the vertebral body, ventral to the pedicle base. The overall 'lifting' force of the device is distributed bilaterally along both the superior and inferior struts, rather than only in the center. Basic Illustration Courtesy: Stryker, Inc

The implants come, in a closed position, in three diameters, 4.2, 5.0, and 5.8 mm. When the devices are opened, size varies from 12.5 to 20 mm in vertical height. The calculated force characteristics show there is a doubling in Newton strength from the 4.2 to 5.8 mm size device, providing both internal support of the the fractured endplate and osteoporotic vertebra but also able to support weight load above the implants [13] (Figure 2).

**Figure 3:**
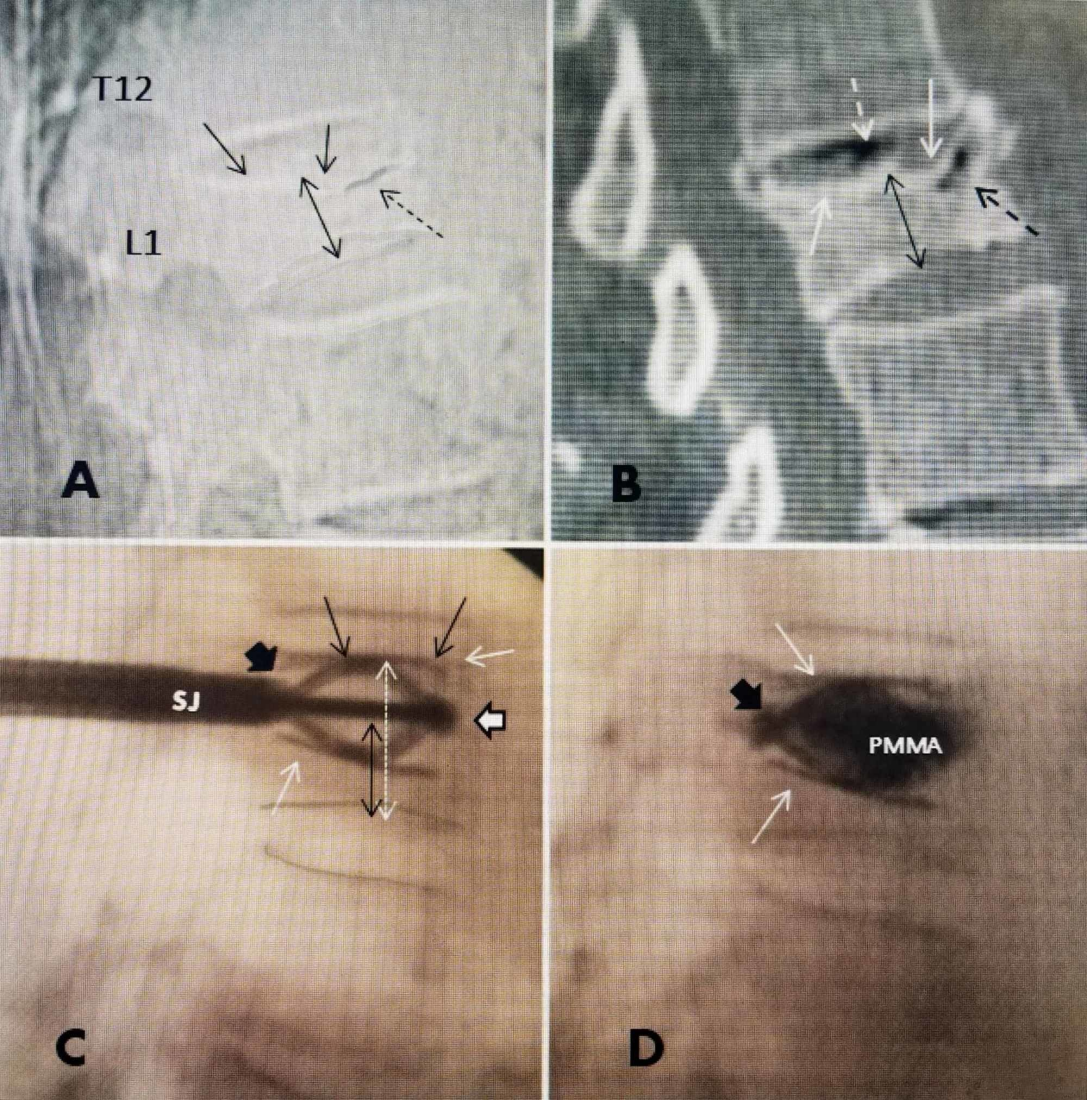
Comparison of preoperative lateral radiograph, sagittal CT scan, and intra-operative films after SpineJack deployment, both before and after cement injection, demonstrating restoration of height of L1 fracture by the implant. A: Lateral plain radiograph showing superior endplate collapse at L1 (solid black arrows) and anterior superior vertebral cleft in the vertebral body just under the fractured endplate (dashed black arrow). The mid-vertebral collapse is marked showing 50% collapse of L1 (double-headed black arrow). B: Sagittal CT scan with bone window demonstrating vacuum cleft in T12-L1 disc space (dashed white arrow) with an additional small vacuum cleft, that can be a sign of osteonecrosis, anteriorly and superiorly in T12 (dashed black arrow). The mid-vertebral height is marked (double-headed fine dashed arrow) indicating almost 50% compression from the collapse of both superior and inferior endplates. C: Lateral intra-operative fluoroscopic film showing the SJ inserted and deployed before the cement is injected. The implant insertion tip is seen anteriorly (solid large white arrow with black border). The struts or wings are deployed and pushing up under the superior endplate (solid black arrows). The inferior strut is restoring support inferiorly (solid white arrow). The original compressed height of the mid-vertebra is indicated (double headed dashed black arrow) compared to the expanded height (double-headed white arrow). D: Lateral film after cement (PMMA) injected through SJ, and the cement is seen to be mainly confined within the boundaries of the implant, with minor spread posteriorly along the connection, just ventral to the base of the pedicle, which is between the insertion handle and the implant (solid large black arrow). The fully extended inferior and superior struts can be seen (solid white arrows) in the same position as before cement injection in 'C'. The four struts are able to broadly re-distribute the load along both superior and inferior endplates and combined with midline PMMA, effectively across the endplates.

In this case, two 5.8-mm-diameter SpineJack^R ^ devices were implanted using a percutaneous, minimally invasive posterior surgical approach, at an outpatient surgical center, using local anesthesia with minimal sedation. All surgical tools are supplied with the device [10]. With the patient being in a prone position, the device was inserted into the fractured L1 vertebra through the pedicle. The implant was then expanded using a specially designed tool which locks into the device and pulls the two ends of the implant towards each other, deploying the struts under the endplates. This compression of the device causes the implant to open in an inferior-superior direction due to the machined threads. A simple mechanism locks the implant into the desired position controlled by the physician. Once the desired expansion was obtained, the device remains in place inside the restored vertebra and polymethylmethacrylate (PMMA) bone cement was injected through the center of the the device via an attached pipette which has side ports to allow flow of cement outside of the device. Cement initially flows through an aperture in the anterior third of the center of the device and goes initially, anterior and medial, providing broad support to the fractured superior endplate and then slowly fills dorsally. Regular fluoroscopic imaging throughout the operative procedure ensures correct implantation and location directly under the fracture before cement is injected and monitors the position of the cement as it is injected (Figure 3).

## Discussion

**Figure 4:**
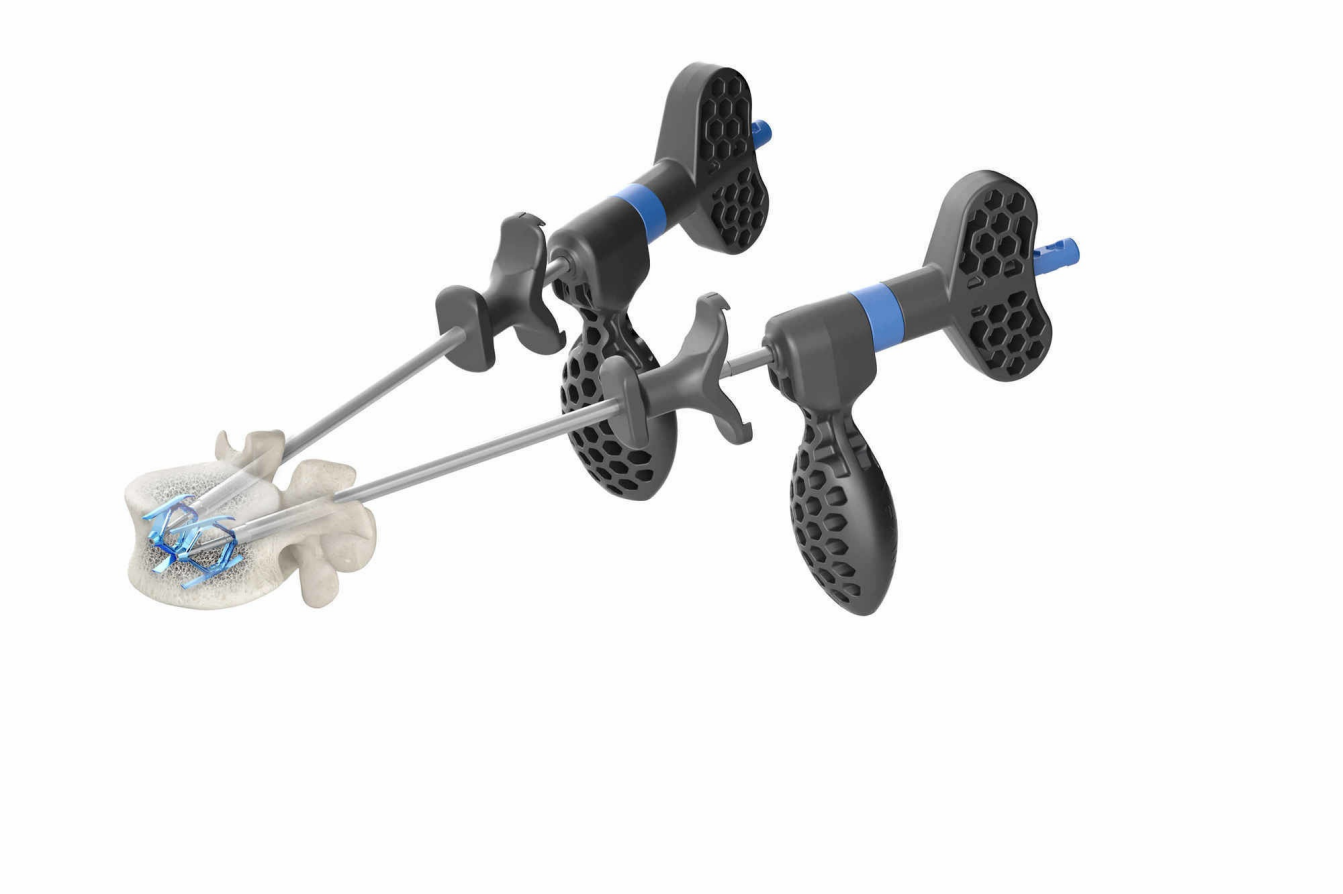
Illustration of two SpineJack implants in place after expansion but before cement inserted The specifically sized implants are inserted transpedicular, similar to percutaneous pedicle screws, centered under the depressed endplate fracture. Once deployed under fluoroscopic guidance, the downward positioned detachable oblong handles are exactly oriented to the position of the SpineJack in the sagittal plane. Picture Courtesy: Stryker, Inc

The thoracic-lumbar junction is anatomically defined as encompassing the vertebrae from T11 to L2 and specifically the adjacent segments at T12 and L1 which is the region of transitional movement between the relatively immobile thoracic spine with the mobile lumbar spine [1]. One of the major issues with fractures at the thoraco-lumbar junction is the increased incidence and risk of progressive vertebral collapse or even vertebra plana, defined as greater than 70% loss of vertebral height, as well as the gradual development of kyphotic deformity with increased risk of adjacent level fractures [2,7-8]. Vertebral augmentation (VA) and balloon kyphoplasty (BKP) with polymethylmethacrylate cement (PMMA) have been the standard approaches to correcting the collapse and deformity [5-7]. VA directly fills the vertebra with cement but BKP was felt to offer the advantage of expansion of the collapsed endplate by the balloon [5-6,9]. The initial intra-operative expansion was caused by expanding different size balloons on one or both sides, then removing the balloon and injected PMMA to 'maintain' the resultant correction in vertebral height. However, multiple follow-up studies have consistently demonstrated loss of height as soon as thirty days and progressing up to twelve months following the procedure. Correction can be lost in 10-30% of cases in followup with subsequent risk of progressive kyphosis [8-10]. Between 10% and 25% also developed fractures at the next adjacent, usually superior osteoporotic vertebra, and a number of studies found the incidence was actually higher after BKP compared to VP [6-7]. The biomechanical reason for adjacent level fractures above vertebral fractures has been proposed that the kyphosis and spinal angulation leads to shifting of the center of gravity more anteriorly, plus the secondary stress effects on osteoporotic bone of large amount of globular bone cement, which both make the treated vertebra more rigid and put stress on the adjacent osteoporotic level above [10-11]. Understanding that there was nothing in the vertebra to provide an underlying structural framework to maintain the correction, the concept of using a permanent implant to maintain the initial height correction evolved with use, initially of polyetheretherketone (PEEK) 1-mm stackable wafers, and later a variable expandable spiral implant combined with subsequent injection of PMMA cement [14-15]. The Spinejack^R^ has evolved from these previous concepts to both expand and maintain initial vertebral height correction by using bilateral titanium implants that provide both para-lateral support to the fractured vertebrae and endplates in line with the pedicle, combined with central and anterior vertebral support as the cement interdigitates between the two devices [13]. The unique expandable design of the 'struts' on the implant effectively spread the load, providing broad, more diffuse support under the depressed and fractured endplates [13]. The permanent implants significantly decrease the possibility of the loss of initial height correction seen after BKP, as well as the risk of recurrent collapse, kyphosis, and adjacent level fractures (Figure 4). 

**Figure 5:**
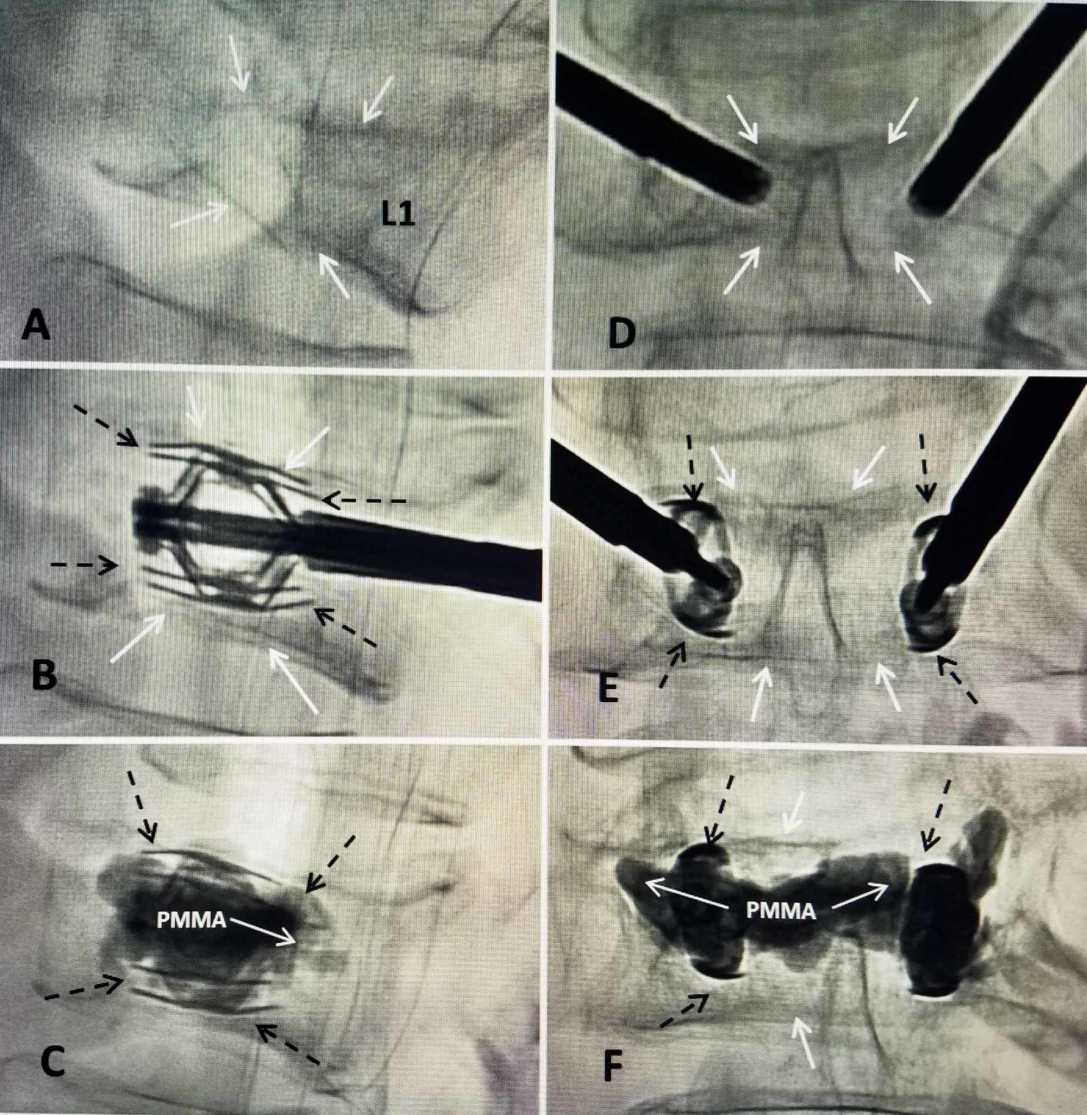
T12 superior endplate collapse with greater than 50% collapse, reconstituted with bilateral expansion of two SpineJack devices under the endplate followed by cement augmentation to maintain the correction A: Lateral intra-operative digital fluoroscopy showing the collapse of both superior (two solid white arrows) and inferior endplates (single solid white arrow) of T12. B: Lateral film after partial insertion and expansion of bilateral 5.0 mm SpineJack implants. The struts of the expanded device are seen (dashed black arrows). The post-procedure films show re-expansion of both endplates (solid black arrows) leading to restoration of mid-vertebral height. C: Lateral film after injection of bone cement (PMMA) through the center channel of the device. The deployed struts under the previous compressed superior endplate can be seen (dashed black arrows). D: Anterior-posterior intra-operative film showing both SpineJack devices inserted before expansion. The collapsed endplates are seen (solid white arrows). E: Symmetric expansion of the SpineJack before cement injection. There is restoration of the vertebral height, especially in the center, with associated elevation of both endplates (solid white arrows). The struts or 'wings' of the SpineJack can be seen (dashed black arrows). F: Anterior-posterior film after completion of cement injection. The cement (PMMA) is seen surrounding and within both SpineJack implants, and crossing the midline between the two implants (solid white arrows). The edges of the struts are marked containing the PMMA (dashed black arrows) and can be compared to lateral view in image 'C'.

Obtaining and especially maintaining height correction of the vertebral collapse has been a problem with balloons since after the balloon is removed, the cement that is injected is assumed to maintain the height correction [6,8-10,12]. Different size and shape balloons going from 10.0 x 2.0 mm to 20.0 x 5 mm have been developed to create bigger holes in the fractured vertebra and then filling with cement, resulting in the use of up to 4 to 6 cc of cement [7-9]. This has lead to the risk of increased leakage both into the adjacent disc space and the spinal canal [6-7]. Studying the pathologic anatomy of an osteoporotic compression fracture, the balloon is basically taking a compressed 'eggshell' like fracture and creating a larger 'hollow' defect within the already weakened vertebral body with the balloon, which is then filled by more denser cement now in a more concentrated location [1,11]. Paradoxically, this creates more rigidity of the vertebral body, possibly affecting the softer adjacent osteoporotic vertebral bodies leading to further fractures [7-10]. With the SJ implants, by leaving the thin titanium implant in place, the endplates are restored to a more normal height which is maintained by the implant itself that is then supplemented by bone cement that flows around the implant, filling initially from the most anterior and central area then to more posterior [13]. These titanium implants could also be used as a structural adjunct in treating fractures related to destructive vertebral tumors combined with radiation or intratumoral therapy. In a 36-month follow-up study comparing BKP to SJ, clinical improvements were observed with both procedures over the three-year period, vertebral height restoration/kyphotic correction was still evident at 36 months, with a greater mean correction of anterior (10 ± 13% vs 2 ± 8% for BKP, *p *= 0.007) and central height (10 ± 11% vs 3 ± 7% for BKP, *p* = 0.034) and a larger correction of the vertebral body angle (− 5.0° ± 5.1° vs 0.4° ± 3.4°; *p* = 0.003) for SJ group [16-18]. The initial correction is both maximized and maintained while using less PMMA, an average of 2.5 to 3.00 cc of cement in total, leading to less risk of cement leakage (Figure 5).

The prevailing biomechanical explanation for the increased risk of adjacent fractures above osteoporotic compression fractures, in general and possibly increased after BKP, is that combined with the increased density and rigidity of the treated vertebra after BKP, and associated with the shifting of the center of gravity more anteriorly with deformity and kyphosis, places more load on the superior and anterior adjacent less dense osteoporotic vertebra leading to further fractures, ranging from 20 to 30% in some series [11]. Different researchers have noted, using pressure studies on the adjacent disc above a compression fracture, that the problem of adjacent level fractures after VA and BKP may actually be related to loss of supporting pressure in the supra-adjacent disc, as the disc 'expands' into the collapsed vertebra which then effects in poorer support and weight bearing for the vertebra above [14-16]. Depression of the fractured endplate alters the pressure in the disc, thereby increasing the load that gets transferred to the anterior wall of the often osteoporotic, adjacent vertebra, by increasing the compressive forces distributed to that vertebra, making it more prone to collapse. Studies in thoracolumbar specimens that have had a balloon cavity formation and cement injected under the fractured endplate have shown that disc pressure decreased during flexion in discs with fractured endplates, as opposed to discs with un-fractured endplates that demonstrated disc pressure that increased during flexion [16-17]. It has been shown that the previous PEEK implants restored pressure in the affected disc associated with vertebral endplate fracture and may be related to the significant lower incidence of adjacent level fractures seen in long term SJ studies [14-15]. More recent intra-operative pre and post SJ implant studies show restoration of the adjacent superior disc pressures closer to normal which correlates with a lower incidence of adjacent level fractures compared to BKP [13,17-18]. 

## Conclusions

This case of the development of an L1 lumbar osteoporotic fracture, above a previous interlaminar stabilization at L4-5, with signs of fracture instability was treated by using the SpineJack^R ^ vertebral implants combined with bone cement. It was possible to not only restore vertebral height closer to normal, but also obtain better spinal alignment and which significantly reduces the risk of later kyphotic deformity. Long-term follow-up studies using these implants, compared to balloon kyphoplasty, have demonstrated that the initial height and angulation corrections are better maintained, especially in the anterior and middle vertebral body, and is associated with lower long-term spinal pain, and most importantly a finding of less adjacent level fractures. The use of internal structural supports combined with PMMA has a sound biomechanical basis by providing anterior and bilateral structural load support in line with the pedicles. There are two factors that are important in treating these high-risk fractures, lessening the anterior shift of the center of gravity from spinal deformity and secondarily restoring adjacent disc pressure both contributing to less risk of later adjacent level osteoporotic fractures. 
